# Maternal and paternal obesity differentially reprogram the ovarian mitochondrial biogenesis of F1 female rats

**DOI:** 10.1038/s41598-023-42468-5

**Published:** 2023-09-19

**Authors:** Amina G. Ramadan, Wafaa M. Abdel-Rehim, Rasha A. El-Tahan, Samar S. Elblehi, Maher A. Kamel, Sara A. Shaker

**Affiliations:** 1 Department of Biochemistry, Medical Research Institute, Alexandria University, 165 El-Horreya Avenue, EL-Hadara, POB: 21561, Alexandria, Egypt; 2 Department of Pathology, Faculty of Veterinary Medicine, Alexandria University, Alexandria, Egypt

**Keywords:** Biochemistry, Molecular biology

## Abstract

Obesity has harmful consequences on reproductive outcomes and the rapid increase in obesity is assumed to be influenced by epigenetics and trans-generation effects. Our study aimed to explore the effect of maternal and/or paternal obesity on the ovarian tissues of the first-generation female offspring in rats. The study was conducted on 40 adult Wistar albino rats (20 males and 20 females). Obesity was induced by feeding them an obesogenic diet for 3 months. The pregnancy was induced in the females by mating with males in four combinations: healthy mother with healthy father (control parents, CP), healthy mother with obese fathers (OF), obese mothers with healthy father (OM), and obese mother with obese father (obese parents, OP). After delivery, the female offspring at two months were sacrificed, and the blood and ovarian tissues were collected to assess the studied parameters. Our result showed differential impacts of maternal and paternal obesity on the ovarian health of the female offspring. The female offspring of obese OM or OP showed early signs of obesity. These metabolic abnormalities were associated with signs of ovarian lesions, impaired folliculogenesis, and decreased oocyte quality and also showed significant alterations in mitochondrial biogenesis, redox status, inflammation, and microRNAs expression (miR-149 and miR-494). In conclusion, altered ovarian expression of microRNAs and associated impaired mitochondrial biogenesis pathways may be the root causes for the observed intergeneration transmission of the obesogenic phenotype.

## Introduction

The energy imbalance between calories ingested and calories gained is the main cause of the major public health issues known as overweight and obesity. Numerous interconnected factors, including diets, inactivity, sociodemographic traits of the family, environmental factors, and hereditary factors, contribute to obesity. According to the World Health Organization (WHO) in 2016, more than 1.9 billion adults and about 400 million children and adolescents were overweight or obese, affecting more than one-third of the world's population, and that number of obese newborns and children is expected to reach to 70 million by 2025^1^. Obesity is one of the main risk factors for cardiovascular, metabolic, respiratory disorders, and cancer^2^.

The impact of obesity on the individual's health may extend to affect the health of the offspring of obese individuals which increases the social and economic burden to individuals, families, and the healthcare system. Children are more likely to become obese if one or both of their parents are obese: the likelihood of becoming obese increases from 30 to 90% depending on which parent is obese and childhood obesity is linked to a mother's preconception of body mass index (BMI) of 30 kg/m^2^, an abnormal pregnancy weight gain, and gestational diabetes mellitus^3^.

Obesity has well-documented harmful consequences on reproductive outcomes. The obese females developed morphological changes in their reproductive organs, including the uterus, ovary, and oviduct, as well as lipid droplet accumulation^4^. The ovary is a highly organized composite of germ cells (oocytes or eggs) and somatic cells (granulosa cells, thecal cells, and stromal cells), and the interactions between these cells control the development of oocyte-containing follicles, the development of both oocytes and somatic cells as follicles, ovulation, and the formation of the corpus luteum (the endocrine structure that develops from the ovarian tissues^5^. The mature oocytes contain a superior number of mitochondria than other mammalian cells to produce energy through oxidative metabolism^6^, so mitochondrial function plays an important role in follicular growth and development and oocyte quality^7^. The inhibition of mitochondrial metabolism and biogenesis could impede follicular growth and maturation^8^. The impaired mitochondrial function and/or biogenesis have been implicated in reproductive ageing and may impact folliculogenesis and increased incidence of aneuploidy^9^.

The mitochondrial biogenesis and functions are dependent mainly on nuclear genes; peroxisome proliferator activated receptor-γ coactivator-1α (PGC-1α), mitochondrial transcription factor A (Tfam), and nuclear respiratory factor 1 (NRF1)^10^. The nuclear expression of PGC-1α stimulates the expression of Tfam and NRF1, which are required for mitochondrial DNA transcription and replication and regulates mammalian mtDNA copy number (mtDNA-CN)^11^. AMP-activated protein kinase (AMPK) is the major factor of PGC-1α activation for a wide range of tissue. PGC-1α phosphorylation is one of the mechanisms of mitochondrial biogenesis activation via AMPK^12^.

MicroRNAs (miR) are short non-coding RNA that act by translational repression and control the expression of about 60% of the nuclear genome^13^. Many types of miRs act as an important regulator of mitochondrial biogenesis and functions like miR-149 and miR-494 through targeting the PGC-1α/Tfam pathway^13,14^.

Since preconception obesity in females and males may have an impact on their fetal development through programming the phenotype rather than the genotype of the fetus, maternal and paternal obesity may impact not only the adult individual but also the progeny. The molecular and epigenetic changes that may occur in the offspring of obese parents need in-depth investigations to understand the molecular mechanism(s) of the intrageneration effects of obesity for proper management and control of the obesity epidemic. So, the present study aimed to explore the epigenetic changes induced in the ovary of the first-generation (F1) female offspring of an obese mother and/or obese father.

## Materials and methods

### Experimental animals

The current protocol was approved by Alexandria University-Institutional Animal Care and Use Committee (AlexU-IACUC, Approval number: AU01222032211). All experiments fulfill the guidelines of the National Institutes of Health guide for the care and use of laboratory animals (NIH Publications No. 8023, revised 1978) and the recommendations of Egypt's guide for the care and use of laboratory animals. The study is reported in accordance with ARRIVE guidelines. All efforts were made to curb the distress of rats during the experimental period, by using best practice for commonly used procedures, such as blood sampling and feeding rats with a consistent supply of food and water, and simultaneously clean their cages, which can enormously improve animal welfare.

### Ethical statement

The current protocol was approved by Alexandria University-Institutional Animal Care and Use Committee (AlexU-IACUC, Approval number: AU01222032211). All experiments fulfil the guidelines of the National Institutes of Health guide for the care and use of laboratory animals (NIH Publications No. 8023, revised 1978) and the recommendations of Egypt's guide for the care and use of laboratory animals. All efforts were made to curb the distress of rats during the experimental period, by using best practices for commonly used procedures, such as blood sampling and feeding rats with a consistent supply of food and water, and simultaneously cleaning their cages, which can enormously improve animal welfare.

### Induction of obesity

Obesity was induced in young male and female albino rats by feeding them with an obesogenic diet for 3 months. The composition of the obesogenic diet used in this experiment (per 100 g diet) was 30 g protein (300 kcal), 26.5 g fat (195 kcal lard, 70 kcal corn oil), 36.5 g carbohydrate (105 kcal dextran, 106 kcal corn starch, 140 kcal sucrose), 3 g vitamin mix (30 kcal) and 4 g mineral mix (40 kcal)^15^. Rats that were 20% heavier than the mean weight of the control rats of the same age were considered obese.

### Experimental design

The pregnancy was induced in the females by mating with males (one male and one female per cage) in Four combinations:

- *Group I (Control Parents, CP)*: Included mating between 5 healthy females and 5 healthy males.
- *Group II (Obese Fathers, OF)*: Included mating between 5 healthy females and 5 Obese males.
- *Group III (Obese Mothers. OM)*: Included mating between 5 obese females and 5 healthy males.
- *Group IV (Obese Parents, OP)*: Included mating between 5 obese females and 5 obese males.

All the obese female rats were maintained under the obesogenic diet throughout the experiment period. After normal delivery of all pregnancies, all females are allowed to lactate their offspring (F1). After weaning, all the female rat offspring were fed a normal diet, and their weights were monitored for eight weeks after birth.

### Collection of samples

At the age of two months, The F1 female offspring were sacrificed under deep anesthesia using isoflurane with concentration of 100% to obtain blood and ovarian tissues. The ovarian tissues were washed, one ovary from each rat used for histopathological analysis and the other ovaries divided into three aliquots: the first one, used for DNA isolation for the assessment of mitochondria DNA copy number (mtDNA-CN), second one used for total RNA extraction for quantitative real-time polymerase chain reaction (qPCR) of peroxisome proliferator-activated receptor gamma coactivator-1 alpha (PGC-1α), nuclear respiratory factor-1 (NRF-1), mitochondrial transcription factors-A (Tfam), AMP-activated protein kinase (AMPK), nuclear factor kappa B (NF-κB), nuclear factor erythroid 2 related factor 2 (Nfe2l2), miR-149-5p , miR-494-3p , estrogen receptor alpha (ERα) and estrogen receptor beta (ERβ), and the third aliquot was homogenized in phosphate-buffered saline (0.1 M, pH 7.4) in ratio1:9 and centrifuged at 10,000×*g* for 10 min at 4 °C and the supernatant was stored in aliquots for subsequent determinations of total protein level by Lowry method, malondialdehyde (MDA), and total antioxidant capacity (TAC) by colorimetric methods, and PGC-1α, and p-AMPK protein contents by ELISA.

### Serum parameters

Serum levels of fasting blood glucose (FBG), triglycerides (TG), total cholesterol (TC), and high-density lipoprotein-cholesterol (HDL-C) were assayed using commercially available kits (Bio-Med Diagnostic INC, USA). Low-density lipoprotein-cholesterol (LDL-C) was estimated according to Friedewald's equation, LDL-C (mg/dl) = TC − (HDL-C) − (TG∕ 5). All procedures were performed according to the manufacturer’s instructions. The homeostasis model assessment index for insulin resistance (HOMA-IR) was then calculated using the following formula^16^.

$${\text{HOMA - IR}} = \frac{{{\text{Fasting}}\,{\text{insulin}}\left( {\frac{{\upmu {\text{IU}}}}{{{\text{ml}}}}} \right) \times {\text{Fasting}}\,{\text{glucose}}\left( {\frac{{{\text{mg}}}}{{{\text{dl}}}}} \right)}}{22.5 \times 18}$$

### Sample Preparation for histopathological observations

The ovarian tissue specimens were collected from different rat groups, rinsed in saline solution, and then immediately fixed in 10% buffered formalin (pH 7.4) for at least 24 h. The fixed tissue specimens were processed through the conventional paraffin embedding technique^17^. The paraffin Sections (4.5 μm) were dewaxed and stained with Mayer's hematoxylin and eosin (H&E) stain. Stained sections were examined by light microscope and photographed using a digital camera (Nikon Corporation Co., Ltd., Japan).

The scoring system offered by Sağsöz et al.^18^ was used for histopathological evaluation of the ovarian tissues. The histological sections were examined for the presence of Congestion, oedema, and loss of cohesion (separation of parenchymal cells along with normal ovarian cortex and follicles). The changes were scored from 0 to 3 according to their severity, where 0 represents no pathological finding, and 1, 2, and 3 represent pathological findings of < 33%, 33–66%, and > 66% of the ovary, respectively. The scores for each parameter were summed and the total tissue damage scores were calculated. for quantification of ovarian follicular reserve. Differential follicle counts were made where the number of follicles at various developmental stages per unit area (mm^2^) of the ovarian cortex, was counted^19^. The follicles were classified as primordial, primary, secondary, and antral follicles based on their structural features and they were counted in every tenth section. Only follicles containing an oocyte were counted to avoid counting any follicle twice. Follicles were classified as follows: primordial follicle, oocyte surrounded by a single layer of squamous granulosa cells; primary follicle, intact enlarged oocyte with a visible nucleus and one layer of cuboidal granulosa cells; secondary follicle, more than one layer of cuboidal granulosa cells without antral space; antral follicles (including preovulatory follicles), emerging antral spaces^20^.

### Determination of malondialdehyde (MDA) as thiobarbituric acid reactive substances (TBARS)

Malondialdehyde was determined according to the method of Draper and Hadley. The tissue samples are heated with thiobarbituric acid (TBA) at low pH. The resulting pink chromogen has a maximal absorbance at 532 nm^21^.

### Determination of total antioxidant capacity by ferric reducing antioxidant power

The Ferric reducing antioxidant power (FRAP) is a measure of ''Antioxidant Power''.The method described measures FRAP at low pH, when a ferric-tripyridyltriazine Fe (III)-TPTZ complex is reduced to ferrous Fe (II) form, an intense blue colour with an absorption maximum at 593 nm develops^22^.

### Tissues protein levels of PGC-1α and p-AMPK by ELISA

The tissue contents of PGC-1α were assayed using a specific rat ELISA kit (MyBioSource, Inc, USA Cat no. MBS2706379) according to the instructions of the manufacturer. Also, the tissues p-AMPK were assayed using specific rat ELISA kits (Lifespan Biosciences, Inc, Catalog No. LS-F36060) according to the manufacturer's instructions. The total protein concentration was determined using Lowry's method.

### Tissues mitochondrial DNA copy number (mtDNA-CN)

In the present study, we used a qPCR assay to estimate the abundance of mtDNA relative to nuclear DNA. After total genomic DNA isolation, we used a specific primer pair for mtDNA sequence (mtDNA) and a primer pair specific for nuclear sequence (PGC-1α) to perform the same number of PCR cycles and calculate the relative mtDNA signal to nuclear DNA signal. The nuclear gene was used to quantify nuclear DNA (nDNA) and therefore normalization of the mtDNA amount per the nDNA of the diploid cells using the equation:

$${\mathbf{R}} = {\mathbf{2}}^{{ - {\mathbf{\Delta Ct}}}} \quad {\text{where}}\quad {\mathbf{\Delta Ct}} = {\mathbf{Ct}}_{{{\mathbf{mtDNA}}}} - {\mathbf{Ct}}_{{{\mathbf{nDNA}}}}$$

The total DNA was isolated from the different tissues using a DNeasy kit (Qiagen, USA) according to the manufacturer's instructions. The used primer pair for mtDNA (NC_040919.1): Forward; AATGGTTCGTTTGTTCAACGATT and Reverse; AGAAACCGACCTGGATTGCTC, the primer pair for the nuclear PGC-1α gene (NM_031347.1): Forward; ATGAATGCAGCGGTCTTAGC, and Reverse; AACAATGGCAGGGTTTGTTC. PCR reactions were carried out using SYBR Green PCR Master Mix (Qiagen, Germany), 0.5 μM of each primer pair, and 50 ng genomic DNA under the following conditions: 95 °C for 10 min followed by 40 cycles of 95 °C for 15 s, 60 °C for 30 s and 72 °C for 30 s^23^.

### Gene expression analysis

Thirty mg of the ovarian tissues were used for total RNA extraction using the miRNeasy Mini Kit (Qiagen, Germany) according to the manufacturer's instructions and the concentration and integrity of extracted RNA were checked using nanodrop. The reverse transcription of the extracted RNA was performed using Reverse transcription (RT) was performed by TOPscript™ RT DryMIX kit (dT18/dN6 plus) (Enzynomics, Korea) according to the manufacturer's instructions. The tissue expression of PGC-1α, Tfam, AMPK, NRF-1, Nfe2l2, ERα, ERβ, NF-κB, miR-149-5p and miR-494-3p were quantified in the cDNA by CFX Maestro™ Software (Bio-Rad, USA) using QuantiNova™ SYBR® Green PCR Kit (Qiagen, Germany). Quantitative PCR amplification conditions were adjusted as an initial denaturation at 95 °C for 10 min and then 45 cycles of PCR for amplification as follows: Denaturation at 95 °C for 20 s, annealing at 55 °C for 20 s and extension at 70 °C for 15 s^23^. The housekeeping gene 18S rRNA was used as a reference gene for normalization and U6 was used as a reference gene for miRNAs. The primers used for the determination of rat genes are presented in Table 1. The relative change in mRNA expression in samples was estimated using the 2^−ΔΔCt^ method.

**Table 1:** Primer sequences used for assessment of mRNA gene expression.

Gene	Accession number	Primer sequence	
18s rRNA	NR_046237.2	F:	GTAACCCGTTGAACCCCATT
		R:	CAAGCTTATGACCCGCACTT
PGC-1α	NM_031347.1	F:	GTGCAGCCAAGACTCTGTATGG
		R:	GTCCAGGTCATTCACATCAAGTTC
NRF-1	NM_001100708.1	F:	TTACTCTGCTGTGGCTGATGG
		R:	CCTCTGATGCTTGCGTCGTCT
Nfe2l2	NM_017008.4	F:	CGAGATATACGCAGCAGGAGAGGTAAG
		R:	GCTCGACAATGTTCTCCAGCTT
AMPK	NM_023991.1	F:	GTGGTGTTATCCTGTATGCCCTTCT
		R:	CTGTTTAAACCATTCATGCTCTCGT
NF-κB (P65)	NM_199267.2	F:	CAGGACCAGGAACAGTTCGAA
		R:	CCAGGTTCTGGAAGCTATGGAT
ERα	NM_012689.1	F:	ATGAGAGCTGCCAACCTT
		R:	AACAAGGCACTGACCATC
ERβ	NM_012754	F:	AGGTGCTAATGGTGGGACTG
		R:	ACTTTCTGCCTCCTGGTTTG

### Ethics approval

The study was approved by Alexandria University-Institutional Animal Care and Use Committee (AlexU-IACUC, Approval number: AU01222032211).

## Results

### The body weight during the follow-up period

Results showed a statistically significant increase in body weight in female offspring of obese mothers and obese parents compared with female offspring of control parents during the follow-up period from birth to week eight. Results showed a significant increase in body weight in female offspring of obese fathers compared with those of control parents at the 6th and 7th week of age. However female offspring of obese mothers and obese parents showed a significant increase in body weight compared with female offspring of obese fathers during the follow-up period from 1st week of age until the 5th week. At the age of 8 weeks, the offspring of obese parents showed significantly higher weights compared with the offspring of obese fathers (Table 2).

**Table 2:** The body weight (g) during the follow-up period.

Groups	F1-CP	F1-OF	F1-OM	F1-OP
At birth (g)	7.9 ± 0.6	7.5 ± 0.5	14.1^ab^ ± 1.6	15.6^ab^ ± 1.4
Week 1 (g)	15.7 ± 1.8	15.7 ± 2.7	27.2^ab^ ± 1.3	27.3^ab^ ± 1.6
Week 2 (g)	35.3 ± 1.9	35.3 ± 5.0	63.2^ab^ ± 3.5	61.7^ab^ ± 4.8
Week 3 (g)	54.7 ± 4.2	56.3 ± 15.6	74.5^a^ ± 19.8	86.8^ab^ ± 8.9
Week 4 (g)	77.0 ± 4.4	85.2 ± 14.8	111.2^ab^ ± 14.5	115.3^ab^ ± 10.8
Week 5 (g)	105.8 ± 5.4	113.3 ± 23.1	138.7^ab^ ± 16.7	149.8^ab^ ± 13.3
Week 6 (g)	127.8 ± 13.5	136.7^a^ ± 29.4	154.7 ± 12.9	175.3^a^ ± 16.5
Week 7 (g)	157.9 ± 10.0	174.8^a^ ± 17.2	178.5^a^ ± 10.8	186^a^ ± 19
Week 8 (g)	173.8 ± 8.7	178.8 ± 21.3	191.8^a^ ± 7.7	199.1^ab^ ± 7.9

Data are presented as Mean ± SD and *n* = 8.

*F1* first generation female offspring, *CP* control parents, *OF* obese father, *OM* obese mother, *OP* obese parents.

^a^Significantly different compared with offspring of control parents, and ^b^significantly different compared with offspring of obese fathers by ANOVA followed by Post Hoc test (Tukey), at *P* < 0.05.

### Parameters of glucose homeostasis

All female offspring of obese mothers and/or obese fathers have significantly higher FBG, insulin levels and HOMA-IR compared with offspring of control parents. No significant difference was observed between the offspring of obese fathers, obese mothers, or obese parents (Table 3).

**Table 3:** Serum parameters and ovarian redox parameters.

	F1-CP	F1-OF	F1-OM	F1-OP
Serum FBG (mg/dl)	89.8 ± 5.9	153.0^a^ ± 43.4	144.5^a^ ± 33.5	138.6^a^ ± 19.8
Serum Insulin (µIU/ml)	9.1 ± 1.0	16.9^a^ ± 2.2	17.6^a^ ± 0.9	18.8^a^ ± 2.3
HOMA-IR	2.0 ± 0.3	6.2^a^ ± 1.3	6.2^a^ ± 1.2	6.5^a^ ± 1.4
Serum TG (mg/dl)	71.4 ± 8.6	118.7^a^ ± 14.6	171.5^ab^ ± 30.2	167.8^ab^ ± 13.8
Serum TC (mg/dl)	106.5 ± 10.9	118.9 ± 9.8	136.9^ab^ ± 14.2	148.8^ab^ ± 15.9
Serum HDL-C (mg/dl)	59.3 ± 8.5	48.7^a^ ± 3.9	62.0^b^ ± 8.1	62.4^b^ ± 8.2
Serum LDL-C (mg/dl)	33.0 ± 7.2	46.4 ± 8.1	40.6 ± 10.6	53.5^a^ ± 19.4
Serum TAC (µmol/ml)	100.1 ± 5.5	86.5^a^ ± 6.1	81.3^a^ ± 4.7	71.1^abc^ ± 5.3
Ovarian TAC (µmol/mg protein)	6.7 ± 1.6	3.8^a^ ± 0.4	3.9^a^ ± 0.5	3.2^a^ ± 0.6
Ovarian MDA (nmol/gm tissue)	64.0 ± 10.8	80.7 ± 13.4	85.7^a^ ± 14.9	104.5^ab^ ± 19.8

Data are presented as mean ± SD and *n* = 8.

*F1* first generation female offspring, *CP* control parents, *OF* obese father, *OM* obese mother, *OP* obese parents, *FBG* fasting blood glucose, *HOMA-IR* homeostasis model of assessment-insulin resistance index, *TC* total cholesterol, *TG* triglycerides, *MDA* malondialdehyde, *TAC* total antioxidant capacity).

^a^Significantly different compared with offspring of control parents, ^b^significantly different compared with offspring of obese fathers, ^c^significantly different compared with offspring of obese mothers by ANOVA followed by Post Hoc test (Tukey), at *P* < 0.05.

### Parameters of lipid profile

Results showed a significant increase in TG level in female offspring of all obese groups compared with offspring of control parents. Female offspring of obese mothers and obese parents showed significantly higher TG and TC levels compared with offspring of obese fathers. Results showed a statistically significant increase in the TC level of female offspring of obese mothers and parents compared with offspring of control parents. Results showed a significant decrease of HDL-C levels in the female offspring of the obese fathers group compared with the offspring of control parents. Results showed a significant increase in LDL-C level of female offspring of obese parents compared with offspring of control parents (Table 3).

### Redox status parameters

Results showed a significant increase in MDA content in female offspring of obese mothers and obese parents compared with offspring of control parents. Results showed a statistically significant decrease in serum and ovarian TAC in female offspring of all obese groups compared with offspring of control parents. Female offspring of obese parents showed a significant decrease in serum TAC content compared with offspring of obese fathers and obese mothers (Table 3).

### Ovarian expression of Nuclear factor erythroid 2 related factor 2 (Nfe212)

**Figure 1:**
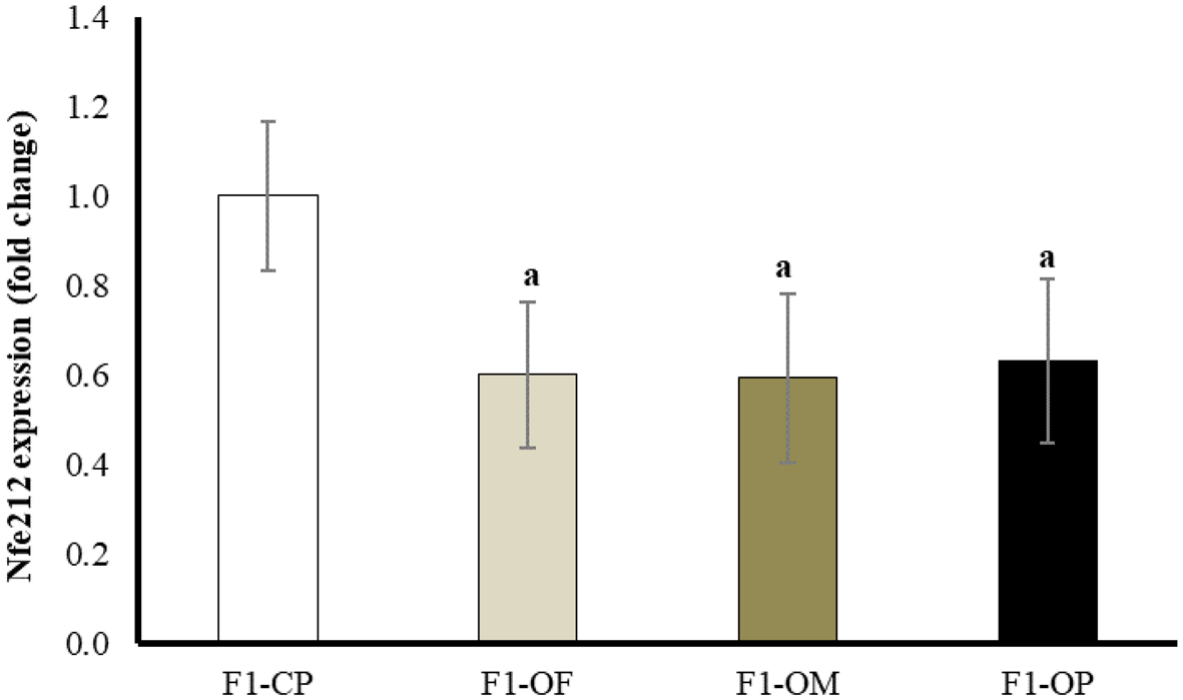
Nuclear factor erythroid 2 related factor 2 expression in different studied groups. Data are presented as mean ± SD and *n* = 8. ^a^Significantly different compared with offspring of control parents by ANOVA followed by Post Hoc test (Tukey), at *P* < 0.05. (Abbreviations: *F1* first generation female offspring, *CP* control parents, *OF* obese father, *OM* obese mother, and *OP* obese parents).

Results showed a statistically significant downregulation of Nfe212 expression in female offspring of all obese groups compared with offspring of control parents. There are no statistically significant differences in Nfe212 expression between female offspring of all obese groups (Fig. 1).

### Inflammatory markers, Ovarian nuclear factor kappa B (NF-κB) expression (Fold change)

**Figure 2:**
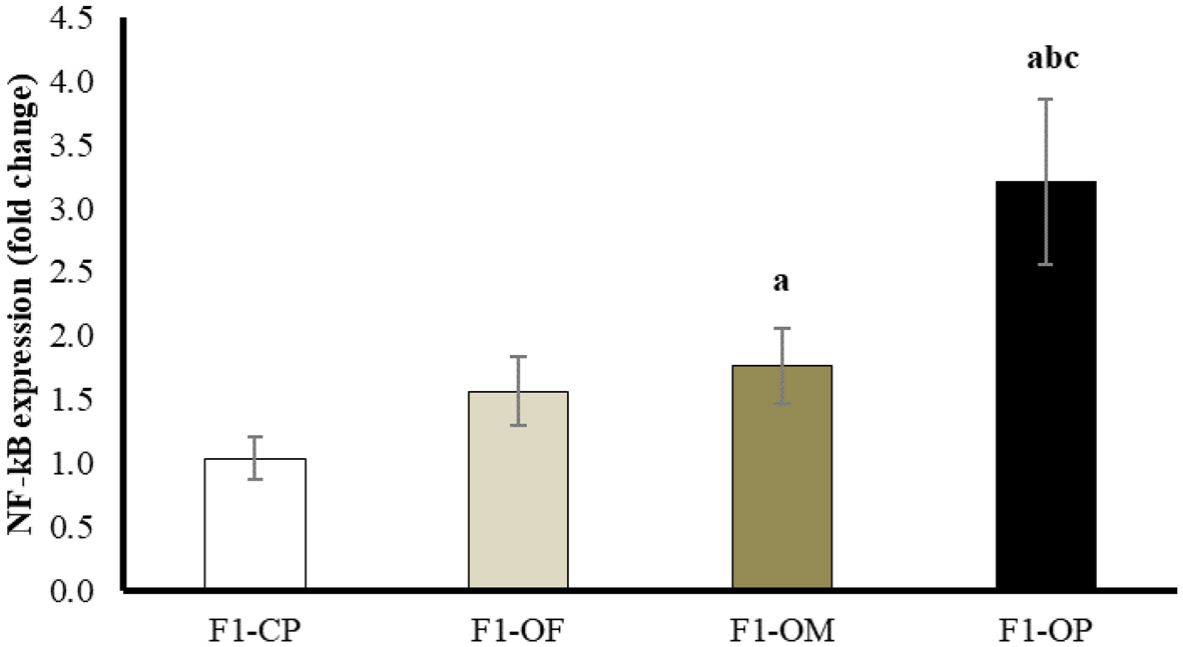
Nuclear factor kappa B expression in different studied groups. Data are presented as mean ± SD and *n* = 8. a: Significantly different compared with offspring of control parents, b: significantly different compared with offspring of obese fathers, c: significantly different compared with offspring of obese mothers by ANOVA followed by Post Hoc test (Tukey), at *P* < 0.05. (Abbreviations: *F1* first generation female offspring, *CP* control parents, *OF* obese father, *OM* obese mother, *OP*; obese parents).

Results showed a significant upregulation of NF-κB expression in female offspring of obese mothers and obese parents compared with offspring of control parents. Female offspring of obese parents showed the highest NF-κB expression compared with other studied groups (Fig. 2).

### Mitochondrial biogenesis parameters

#### Ovarian mitochondrial DNA copy number (mtDNA-CN)/diploid cell

**Figure 3:**
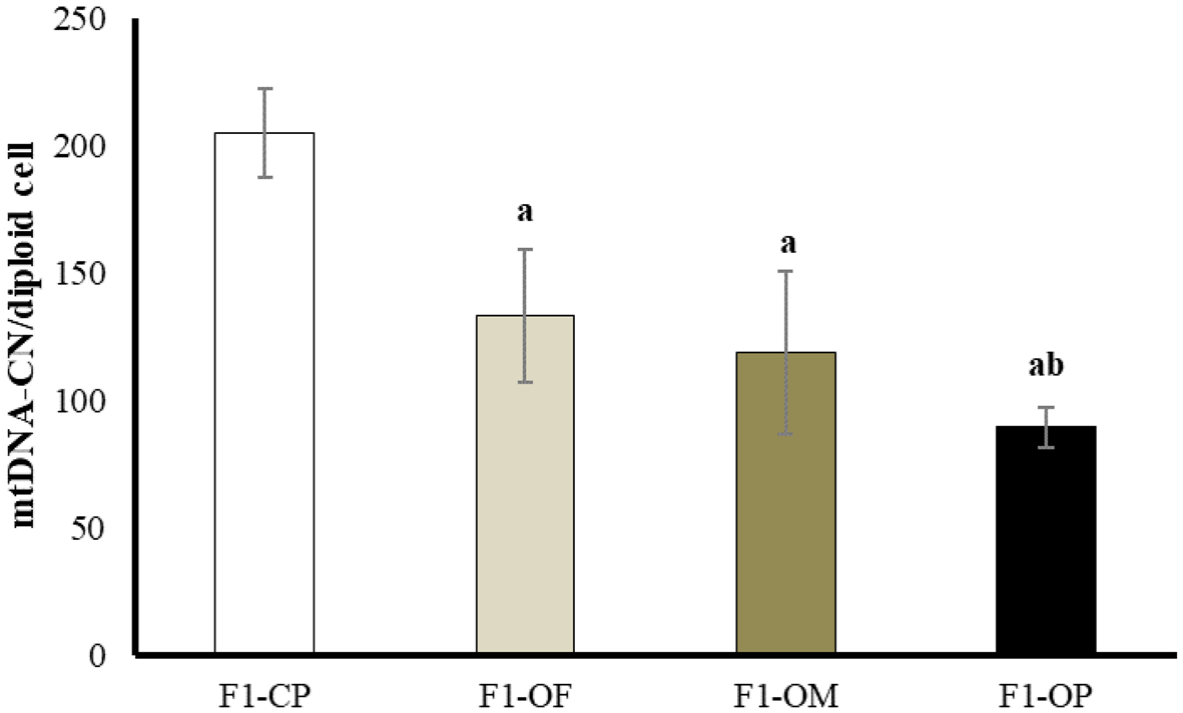
Mitochondrial DNA copy number/diploid cell in different studied groups. Data are presented as mean ± SD and *n* = 8. a: Significantly different compared with offspring of control parents, and b: significantly different compared with offspring of obese fathers by ANOVA followed by Post Hoc test (Tukey), at *P* < 0.05. (Abbreviations: *F1* first generation female offspring, *CP* control parents, *OF* obese father, *OM* obese mother, and *OP* obese parents).

Results showed a significant decrease in mtDNA-CN in female offspring of all obese groups compared with offspring of control parents. Female offspring of obese parents showed a significant decrease in mtDNA-CN compared with offspring of obese fathers. There was no significant difference between the female offspring of obese fathers and the offspring of obese mothers (Fig. 3).

#### Ovarian peroxisome proliferator-activated receptor γ coactivator-1 alpha (PGC-1α) at mRNA expression and protein level

**Figure 4:**
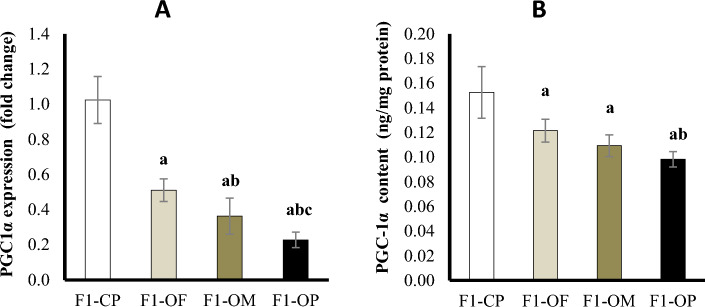
Ovarian peroxisome proliferator-activated receptor γ coactivator-1 alpha (PGC-1α) at mRNA expression (**A**) and protein level (**B**) in different studied groups. Data are presented as mean ± SD and *n* = 8. a: Significantly different compared with offspring of control parents, b: significantly different compared with offspring of obese fathers, c: significantly different compared with offspring of obese mothers by ANOVA followed by Post Hoc test (Tukey), at *P* < 0.05. (Abbreviations: *F1* first generation female offspring, *CP* control parents, *OF* obese father, *OM* obese mother, *OP* obese parents).

At the mRNA level, results showed a significant downregulation of PGC-1α expression in female offspring of all obese groups compared with offspring of control parents. Female offspring of obese parents showed the lowest PGC-1α expression compared with other studied groups. Female offspring of obese mothers showed a statistically significant downregulation of PGC-1α expression compared with offspring of obese fathers (Fig. 4A).

At the protein level, all obese groups showed a significant decrease in PGC-1α content compared with the offspring of control parents. Female offspring of obese parents showed the lowest PGC-1α content compared with other studied groups. There was no significant difference in PGC-1α content between the offspring of obese fathers and the offspring of obese mothers (Fig. 4B).

#### Ovarian AMP-activated protein kinase (AMPK) at mRNA expression and protein level

**Figure 5:**
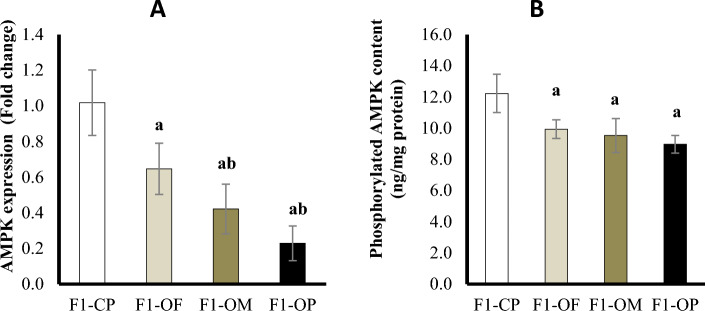
Ovarian AMP-activated protein kinase (AMPK) at mRNA expression (**A**) and protein level (**B**) in different studied groups. Data are presented as mean ± SD and *n* = 8. a: Significantly different compared with offspring of control parents, and b: significantly different compared with offspring of obese fathers by ANOVA followed by Post Hoc test (Tukey), at *P* < 0.05. (Abbreviations: *F1* first generation female offspring, *CP* control parents, *OF* obese father, *OM* obese mother, and *OP* obese parents).

At the mRNA level, results showed a significant downregulation of AMPK expression in female offspring of all obese groups compared with offspring of control parents. Female offspring of obese parents and obese mothers showed significant downregulation of AMPK expression compared with offspring of obese fathers (Fig. 5A).

At the protein level, all obese groups showed significant decrease in p-AMPK content compared with the offspring of control parents. There was no statistically significant difference in p-AMPK content between female offspring of different obese groups (Fig. 5B).

#### Ovarian mitochondrial transcription factors-A (Tfam) and nuclear respiratory factor 1 NRF1 expression (Fold change)

**Figure 6:**
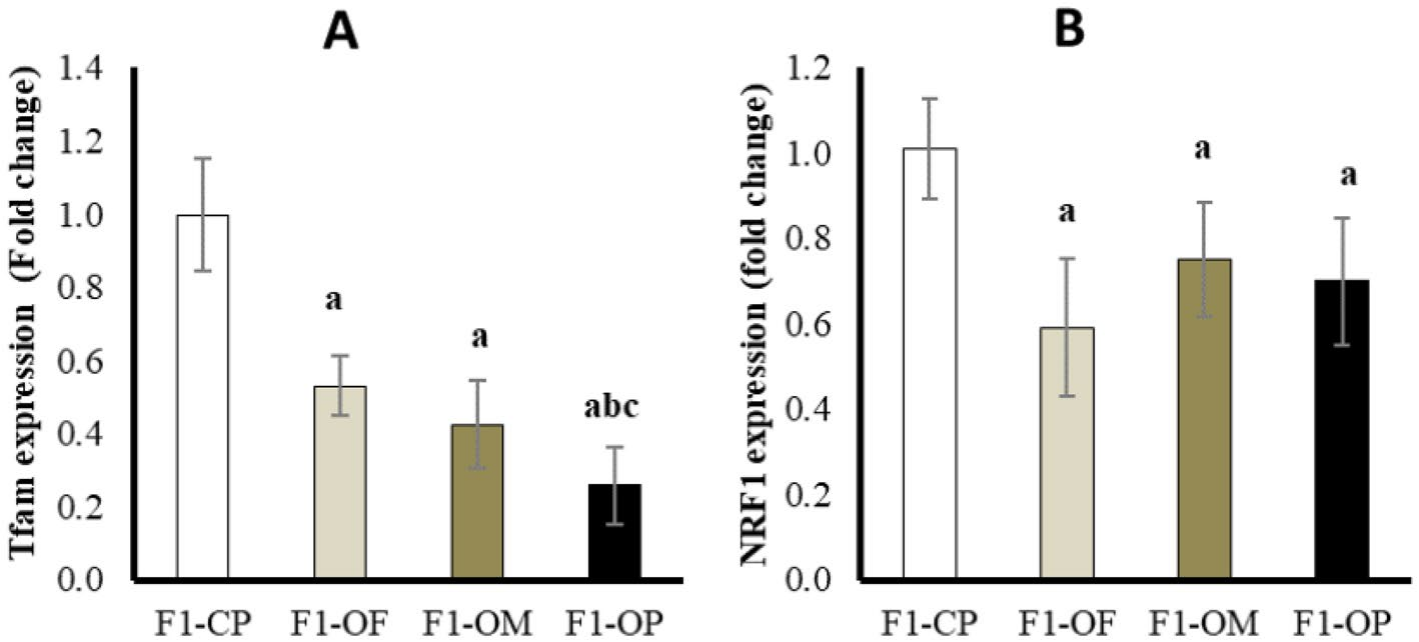
Ovarian mitochondrial transcription factors-A (Tfam) (**A**) and nuclear respiratory factor 1 NRF1 expression (**B**) in different studied groups. Data are presented as mean ± SD and *n* = 8. a: Significantly different compared with offspring of control parents, b: significantly different compared with offspring of obese fathers, c: significantly different compared with offspring of obese mothers by ANOVA followed by Post Hoc test (Tukey), at *P* < 0.05. (Abbreviations: *F1* first generation female offspring, *CP* control parents, *OF* obese father, *OM* obese mother, *OP* obese parents).

Results showed a significant downregulation of Tfam expression in female offspring of all obese groups compared with offspring of control parents. Female offspring of obese parents showed the lowest Tfam expression compared with other studied groups. There was no significant difference between the offspring of obese fathers and the offspring of obese mothers in Tfam expression. (Fig. 6A).

Results showed a significant decrease in NRF-1 expression in female offspring of all obese groups compared with offspring of control parents. There was no statistically significant difference in NRF-1 expression between female offspring of different obese groups (Fig. 6B).

### Ovarian estrogen receptors

**Figure 7:**
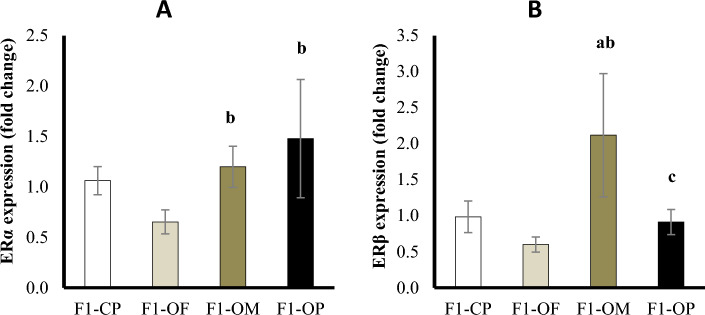
Estrogen receptor α (**A**) and estrogen receptor β (**B**) gene expression in different studied groups. Data are presented as mean ± SD and *n* = 8. a: Significantly different compared with offspring of control parents, b: significantly different compared with offspring of obese fathers, c: significantly different compared with offspring of obese mothers by ANOVA followed by Post Hoc test (Tukey), at *P* < 0.05. (Abbreviations: *F1* first generation female offspring, *CP* control parents, *OF* obese father, *OM* obese mother, *OP* obese parents).

Results showed a significant upregulation of ERα expression in female offspring of obese parents compared with offspring of control parents. Female offspring of obese mothers and obese parents showed significant upregulation of ERα expression compared with offspring of obese fathers (Fig. 7A).

Results showed a significant upregulation of ERβ expression in female offspring of obese mothers compared with offspring of control parents and offspring of obese fathers. Female offspring of obese fathers and obese parents showed no significant difference compared with offspring of control parents. There was a significant decrease in ERβ expression in the female offspring of obese parents compared with the offspring of obese mothers (Fig. 7B).

### Ovarian miR-149-5p and miR-494-3p expression (Fold change)

**Figure 8:**
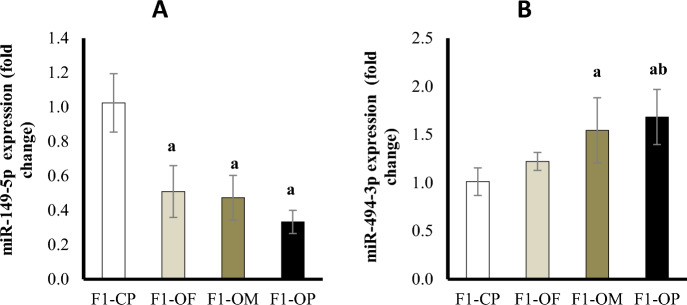
MiR-149-5p (**A**) and miR-494-3p (**B**) gene expression (fold change) in different studied groups. Data are presented as mean ± SD and *n* = 8. a: Significantly different compared with offspring of control parents, and b: significantly different compared with offspring of obese fathers by ANOVA followed by Post Hoc test (Tukey), at *P* < 0.05. (Abbreviations: *F1* first generation female offspring, *CP* control parents, *OF* obese father, *OM* obese mother, and *OP* obese parents).

Results showed a significant downregulation in miR-149-5p expression in female offspring of all obese groups compared with offspring of control parents. There was no significant difference in miR-149-5p expression between female offspring of different obese groups (Fig. 8A). Results showed a significant upregulation in miR-494-3p expression of offspring of obese mothers and obese parents compared with offspring of control parents. There was no significant difference between the female offspring of obese fathers and the offspring of control parents in miR-494-3p expression. There was a significant increase in miR-494-3p expression in the female offspring of obese parents compared with the offspring of obese fathers (Fig. 8B).

### Histopathological examination and histomorphometrical analysis

#### Histopathological findings and lesion scoring

**Figure 9:**
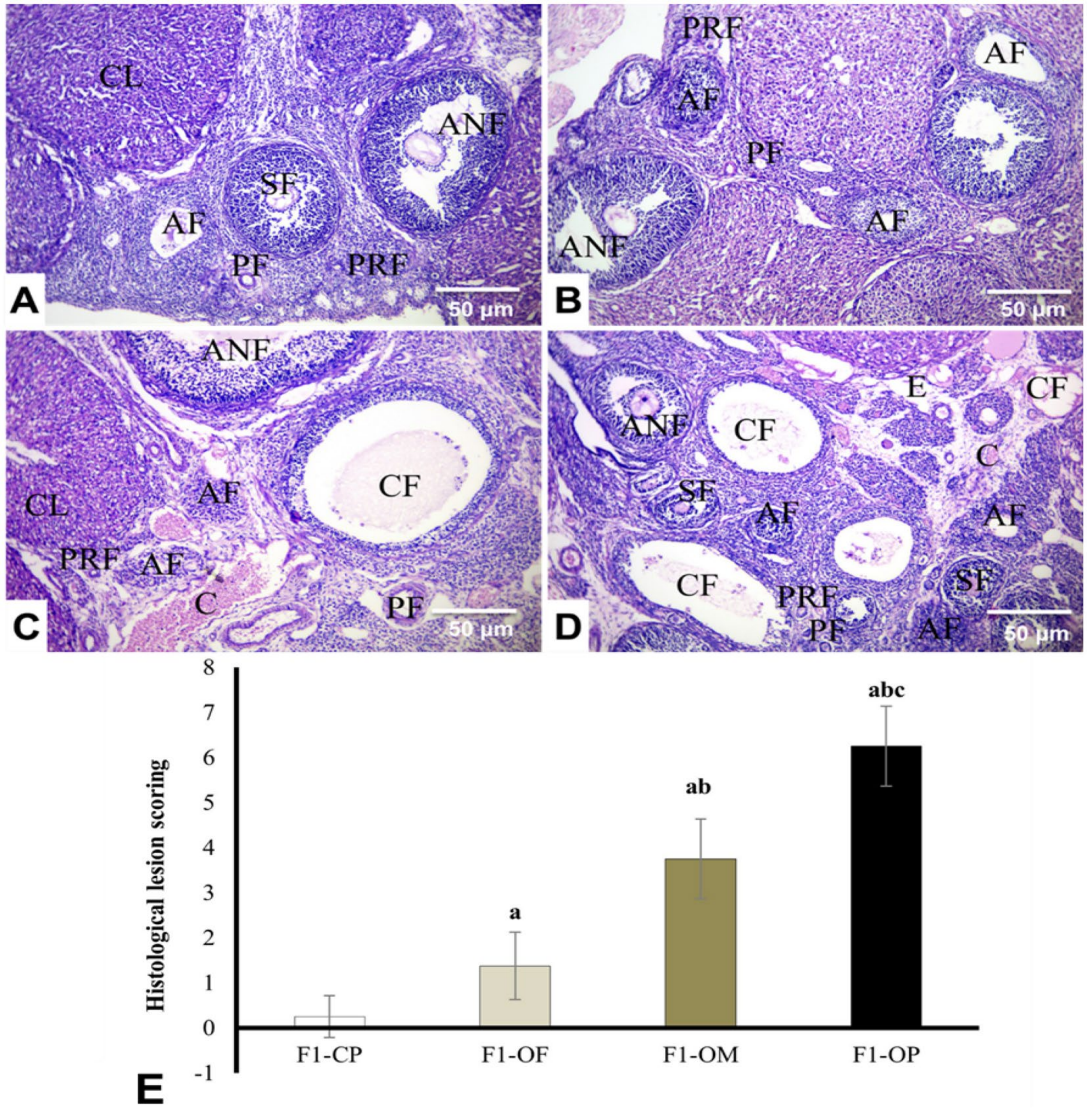
Representative photomicrograph of rat ovaries (HE, × 400). Female offspring of Control parents (**A**), obese father (**B**), obese mother (**C**), and obese parents (**D**). Primordial follicle (PRF), primary follicle (PF), secondary follicle density (SF) antral follicle (ANF), atretic Follicle (AF), corpus lutea (CL), cystic follicles (CF) vascular congestion (C) and edema (E). Quantification of the histological lesion scoring in different studied groups. Data are presented as mean ± SD and *n* = 8. a: Significantly different compared with offspring of control parents, *b*: significantly different compared with offspring of obese fathers, c: significantly different compared with offspring of obese mothers by ANOVA followed by Post Hoc test (Tukey), at *P* < 0.05. (Abbreviations: *F1* first generation female offspring, *CP* control parents, *OF* obese father, *OM* obese mother, *OP* obese parents).

The histopathological examination of ovarian sections of the different experimental groups were shown in Fig. 9A–D. Ovaries of female offspring of control parents showed the typical morphological structures of primary, secondary, and antral follicles (Fig. 9A). The integrity of the ovaries was well preserved in the female offspring of obese fathers (Fig. 9B) and was similar to that of the offspring of control parents. However, ovarian sections of female offspring of obese mothers (Fig. 9C) and female offspring of obese parents (Fig. 9D) showed the presence of numerous degenerated and atretic follicles with basement membrane distortions and separation of theca folliculi from the GCs and thinning of the cumulus oophorous. Vascular congestion, perivascular oedema and mononuclear inflammation in the interstitial tissue were also observed (Fig. 9D).

Results showed a significant increase in histological lesion scoring in female offspring of all obese groups compared with offspring of control parents. Female offspring of obese parents showed the highest histological lesion scoring compared with other studied groups. Female offspring of obese mothers showed higher histological lesion scoring than offspring of obese fathers (Fig. 9E).

#### Histomorphometrical analysis

Results showed a significant reduction in the number of ovarian primary, secondary and antral follicles in female offspring of obese parents compared with offspring of control parents and with offspring of obese fathers. Atretic and cystic follicle numbers showed a significant elevation in female offspring of obese parents compared with offspring of control parents and with offspring of obese fathers. Cystic follicle numbers showed a significant elevation in female offspring of obese parents compared with offspring of obese mothers. Results showed a significant reduction in the number of ovarian primary and antral follicles in female offspring of obese mothers compared with offspring of control parents or of obese fathers. Secondary follicle numbers revealed a significant reduction while that of atretic follicles showed a significant increment in female offspring of obese mothers compared with offspring of control parents (Table 4).

**Table 4:** Histomorphometrical analysis of the ovarian tissues of the F1-female offspring of control parents, obese fathers, obese mothers and obese parents.

Groups	F1-CP	F1-OF	F1-OM	F1-OP
Primordial follicles (n/mm^2^)	18 ± 3	19 ± 4	15 ± 2	13^ab^ ± 4
Primary follicles (n/mm^2^)	13 ± 3	12 ± 3	8^ab^ ± 2	6^ab^ ± 2
Secondary follicles (n/mm^2^)	7 ± 2	6 ± 2	4^a^ ± 2	2^ab^ ± 1
Antral follicles (n/mm^2^)	6 ± 2	5 ± 2	3^ab^ ± 1	2^ab^ ± 1
Atretic follicles (n/mm^2^)	2 ± 1	3 ± 1	3^a^ ± 1	5^ab^ ± 1
Cystic follicles (n/mm^2^)	0 ± 0	0 ± 0	1 ± 1	3^abc^ ± 1

Data are presented as Mean ± SD and *n* = 8.

*F1* first generation female offspring, *CP* control parents, *OF* obese father, *OM* obese mother, *OP* obese parents.

^a^Significantly different compared with offspring of control parents, ^b^significantly different compared with offspring of obese fathers, ^c^significantly different compared with offspring of obese mothers by ANOVA followed by Post Hoc test (Tukey), at *P* < 0.05.

### Correlation studies

The statistical analysis using Spearman 's rank correlation (Table 5) revealed that.

**Table 5:** Correlation studies.

Parameters	Ovarian MDA	Serum TAC	Ovarian NF-κB expression	Ovarian mtDNA-CN	Ovarian AMPK expression	Ovarian miR-149-5p	Ovarian miR-494-3p
Ovarian MDA		− 0.613*	0.411*	− 0.285	− 0.397	− 0.445*	0.066
Serum TAC	− 0.613*		− 0.585*	0.265	0.593*	0.609*	− 0.236
NF-κB expression	0.411*	− 0.585*		− 0.606*	− 0.568*	− 0.432*	0.489*
mtDNA-CN	− 0.285	0.265	− 0.606*		0.422*	− 0.013	− 0.326
AMPK expression	− 0.397	0.593*	− 0.568*	0.422*		0.576*	− 0.509*
PGC-1α expression	− 0.504*	0.771*	− 0.669*	0.436*	0.860*	0.601*	− 0.484*
Tfam expression	− 0.513*	0.528*	− 0.688*	0.492*	0.650*	0.365	− 0.561*
ERα expression	− 0.351	− 0.628*	0.727*	− 0.603*	− 0.483*	− 0.083	0.612*
Ovarian primordial follicle number	− 0.403	0.644*	− 0.383	0.119	0.568*	0.444*	− 0.445*
Ovarian primary follicle number	− 0.293	0.389	− 0.568*	0.426*	0.581*	0.395	− 0.597*
Ovarian secondary follicle number	− 0.374	0.535*	− 0.641*	0.574*	0.391	0.222	− 0.588*
Ovarian antral follicle number	− 0.237	0.562*	− 0.451*	0.418*	0.563*	0.279	− 0.694*
Ovarian atretic follicle number	0.527*	− 0.343	0.589*	− 0.442*	− 0.272	− 0.320	0.262
Ovarian cystic follicle number	0.489*	− 0.738*	0.6*	− 0.43*	− 0.655*	− 0.467*	0.563*
Histological lesion scoring	0.539*	− 0.747*	0.789*	− 0.553*	− 0.652*	− 0.483*	0.505*

*Significant correlation *P* < 0.05.

## Discussion

The intergenerational transmission of obesity is a complex issue that is influenced by many factors including the line of transmission (maternal or paternal), genetic, epigenetic and environmental factors. In the present study, the two-month-old female offspring of the obese mother (alone or combined with the father's obesity) showed early signs of obesity as indicated by heavier body weights, metabolic abnormalities which associated with signs of ovarian lesions, and impaired mitochondrial biogenesis. While the two-month-old female offspring of an obese father showed normal body weight with no or mild signs of ovarian abnormalities and near-normal folliculogenesis, those offspring suffer from significant alterations in mitochondrial biogenesis, which may suggest that those females may develop ovarian abnormalities like those offspring of maternal obesity but at an older age. These differential impacts of maternal and paternal obesity on the ovarian health of the female offspring may be interpreted in the context of the pattern of transmission of the impact and the duration of the impact.

The present results indicated that the female offspring of obese mothers and/or obese fathers showed significant deteriorations in the glucose homeostasis parameters which were associated with significant changes in the serum lipid profile as maternal obesity has the most prominent effects on the serum triglycerides and the paternal obesity has the most prominent effect on HDL-C level, while the parental obesity affects both total and LDL-C levels. The human study indicated that the young offspring of obese mothers have higher glucose and insulin concentrations than those of lean mothers^24^. Maternal obesity has been associated with an increased risk of gestational diabetes, pre-eclampsia, and large birth weight, all of which can lead to health problems for the newborn^25,26^. A study by Zambrano et al. reported that children born to obese mothers had a higher risk of developing obesity, type 2 diabetes, and cardiovascular disease later in life^27^.

The detected metabolic alterations in the female offspring of obese mother and/or father were associated with reproductive changes in the ovarian tissues at different levels, histological, redox, mitochondrial, and molecular one. At the histological level. There are a reduction in the number of all stages of follicles (primordial, primary, secondary, and antral) in the female offspring of obese mothers or obese parents which may indicate a failure in the folliculogenesis that affects oocyte quality and development and may cause decreased fertility of these females. The impaired folliculogenesis is associated with increased numbers of atretic and cystic follicles in the ovarian tissues of female offspring of obese parents and obese mothers with no significant changes in the offspring of obese fathers. The integrity of the ovaries was well preserved in female offspring of obese fathers and was like that of control. However, female offspring of obese mothers or parents showed the presence of numerous degenerated and atretic follicles with basement membrane distortions separation of theca folliculi from the granulosa cell and thinning of the cumulus oophorous. Vascular congestion, perivascular oedema and mononuclear inflammation in the interstitial tissue were also observed. As shown by Yang et al. a high-fat and sugar diet resulted in an increase of early ovarian follicles and a decrease in mature follicles and corpus luteum, Obesity may disrupt normal folliculogenesis through increased production of IL-10 in visceral fats. This relationship may help clarify the reported association between obesity and ovulatory dysfunction, which has been found in patients with polycystic ovary^28^. Parallel to the previous findings a statistically significant increase in histological lesion scoring in female offspring of all obese groups compared with control offspring with the highest histological lesion scoring observed in the offspring of obese parents.

Obesity has a significant impact on folliculogenesis in rats. Studies have demonstrated that obesity can disrupt the normal hormonal balance, leading to alterations in the timing and progression of follicular development^29^. Furthermore, obesity-induced inflammation can also contribute to the disruption of folliculogenesis in rats^30^. It has been demonstrated that maternal obesity changes the levels of insulin, glucose, and free fatty acids in follicular fluid, directly altering oocyte metabolism and slowing oocyte maturation^31^.

At the redox level, the female offspring of obese rats suffer from oxidative stress manifested as an increase in ovarian MDA content (the lipid peroxidation marker), a decrease in ovarian and serum TAC and suppressed ovarian expression of Nfe2l2 (the master regulator of the antioxidant response and redox equilibrium against oxidative stress). All of these may have an impact on the quality of the oocytes^32^. It was found that maternal HFD mediated ovarian oxidative stress in mice offspring as evidenced by increased MDA levels^33,34^. These findings suggest that obesity-induced oxidative stress negatively impacts the oocyte and follicular development, which in turn can affect fertility and pregnancy outcomes confirmed by the positive correlation between MDA level and the number of atretic and cystic follicles, and histologic lesion score. On the other hand, TAC showed a negative correlation with the number of cystic follicles and the histologic lesion score. The obesity-related oxidative stress triggers activation of the NF-κB signalling pathway which promotes target genes transcription, allowing regulation of more inflammatory cytokine production creating a vicious cycle where free radicals production rises and exacerbates oxidative stress^35^. The link between NF-κB and ovarian changes is confirmed by the positive correlation between NF-κB and the number of atretic follicles and histologic lesion score.

At the mitochondrial level, the female offspring of obese mothers and/or fathers showed lower mtDNA-CN compared with the control offspring especially when both parents are obese. Reduced mtDNA-CN in cells results in altered cellular shape, poorer respiratory enzyme function, and reduced production of critical proteins. Besides, dysfunctional mitochondria can no longer provide the metabolic needs of the oocyte^36^. This abnormal pattern of mitochondrial biogenesis is associated with a significant suppression in the ovarian expression of the PGC-1α/Tfam/NRF1 pathway compared to the control showing impaired mitochondrial biogenesis as confirmed by the correlation data indicating the positive association between the mtDNA-CN with the expression of the components of this pathway (PGC-1α, and Tfam). Besides its role as a regulator of cellular antioxidants, Nfe2l2 is essential for controlling mitochondrial activity through interactions with PGC1-1α^37^ which stimulates the production of nuclear respiration factors (NRF1 and NRF2) and estrogen-related receptors, which in turn activate Tfam to work with DNA polymerase and enhance mtDNA replication and increase mitochondrial biogenesis^38^. The enhanced mitochondrial biogenesis and functions are closely linked to follicular growth and development and inhibiting mitochondrial metabolism and biogenesis could impede follicular growth and maturation^8^. This relationship is confirmed in our study by the strong positive correlation between mtDNA-CN with the numbers of primary, secondary, and antral follicles and negative correlations with the numbers of atretic and cystic follicles and histologic lesion score.

At the metabolic level, the ovarian tissues of the female offspring of an obese mother and/or father have a significant decline of AMPK at the mRNA level and the active phosphorylated protein form (p-AMPK) especially when both parents are obese. Obesity is associated with reduced AMPK activation, concomitant with alterations in glycolysis, insulin sensitivity, hepatic lipid metabolism and inflammation^39^. Oriquat et al., 2023 showed that HFD induced significant downregulation of the expression of AMPK and its upstream activating kinase (LKB1), and p-AMPK and LKB1 protein levels^40^. The role of AMPK in ovarian maturation was confirmed in the present study by the correlation data which indicated that AMPK showed a positive correlation with the number of primordial, primary, and antral follicles and negatively correlated with the number of cystic follicles and histologic lesion score. AMPK activation in oocytes can enhance mitochondrial function and biogenesis, reduce oxidative stress and improve embryo quality^41,42^. The link between AMPK and mitochondrial biogenesis was evident in the present study by the strong positive correlation between AMPK and mtDNA-CN and PGC-1α.

Maternal and paternal obesity appear to differentially affect the estrogen signalling in the ovarian tissues of the offspring as the ovarian expression of ERα showed a non-significant decline when the father is obese while showed a non-significant increase when both parents are obese. The expression of ERβ showed significant induction in the offspring of an obese mother only while showing a non-significant decline in the offspring of the obese father. Kuryłowicz showed that obesity is associated with a significant decrease in the expression of both nuclear ER subtypes in adipose tissue, while weight loss leads to an increase in ERα and ERβ mRNA levels^43^. According to Scudiero and Verderame after 4 weeks of HFD, ERβ transcripts are down-regulated, whereas ERα levels remain unchanged; after 12 weeks, both ERα and ERβ expression is up-regulated^44^. These effects need in-depth investigation as obesity can disrupt estrogen signalling pathways in the ovary, leading to conditions such as polycystic ovary syndrome (PCOS)^45^. Obesity is associated with an increased risk of estrogen receptor-positive breast cancer, which is believed to be linked to changes in estrogen signalling in the body^46^ and it was reported that obesity can lead to changes in the expression of estrogen-related genes in ovarian tissue^47^.

At the microRNA level, we found a marked reduction in the ovarian expression of miR-149 in the female offspring of obese mothers and/or fathers. On the other hand, the female offspring of the obese mothers showed significant upregulated ovarian expression of miR-494 while the offspring of the obese fathers showed no significant changes. miR-149 and miR-494 play opposing roles in regulating mitochondrial biogenesis by targeting different transcriptional coactivators, PGC-1α and PGC-1β, respectively. There is limited research on the specific relationship between obesity and miRNA-149 expression in the ovary. However, PGC-1α activity and mitochondrial biogenesis are stimulated by miR-149 and HFD and obesity have been demonstrated to drastically diminish SIRT1 activity by reducing the expression of miR-149^48^. These results imply that miR-149 overexpression could protect against obesity-related endothelium impairment by replenishing SIRT1^49^. The importance of miR-149 in regulating ovarian functions was evidenced by its positive association with several primordial follicles, TAC, PGC-1α, and AMPK and its negative association with ovarian MDA and NF-κB. It has been demonstrated that miR-494 targets the 3′UTR of both PGC-1α and SIRT1, a deacetylase that is also involved in the regulation of mitochondrial biogenesis, so it can inhibit mitochondrial biogenesis and reduce mitochondrial content^50^. The expression of PGC-1α was markedly declined in response to miR-494 overexpression^14^. This finding was confirmed in our study by a negative correlation of mir-494 and PGC1α expressions. The present study indicated that the correlation pattern of miR-494 is opposite to that of miR-149 as miR-494 is negatively correlated with the number of primordial follicles, the number of antral follicles, PGC-1α, and AMPK and positively correlated with NF-κB, histological lesion score, and the expression of ERα.

These differential impacts of maternal and paternal obesity on the ovarian health of the female offspring may be interpreted in the context of the pattern of impact transmission and the duration of the impact. Maternal obesity operates early during the pre-gestational period on the developing ova through epigenetic effects on nuclear genes and impairments of mitochondrial biogenesis and functions that affect folliculogenesis and the quality of the ova. Then after fertilization and during the gestation maternal obesity may impact and reprogram the developing fetus through the obesogenic intrauterine milieu with disturbed metabolites, adipocytokines, and hormones. Also, post-gestation the maternal obesity still affects the newborn baby during lactation through suckling obesogenic milk with high-fat contents. On the other hand, the impact of paternal obesity is limited to the epigenetic effects on the sperm that may induced during the pregestational period.

## Conclusion

These maternal or parental-induced ovarian changes in the female offspring may explained by multiple interrelated mechanisms including obesity microenvironment-induced pregestational and gestational alterations in the maternal ova or the developing embryos, redox imbalance, intrauterine inflammation, disturbed mitochondrial functions and biogenesis, and epigenetic abnormalities. All these obesity-induced abnormalities in the ova may affect the normal development of the embryos obtained from these ova and program the female offspring for obesity and abnormalities in different organs especially the ovary, giving into account the fact that, the number of germline cells (ova) of the female offspring is setup during gestation under the obesogenic milieu of the mother.

## Data Availability

All data available upon your request to the corresponding author.
